# Rethinking Phenotypic Categorization: Evidence From Morphological and Life‐History Traits in Spiny Lizards (*Sceloporus*)

**DOI:** 10.1002/ece3.71709

**Published:** 2025-06-29

**Authors:** Isaac Emmanuell Diaz‐Ortega, José Jaime Zúñiga‐Vega, Oscar Flores‐Villela, Hibraim Adán Pérez‐Mendoza

**Affiliations:** ^1^ Laboratorio de Ecología Evolutiva, Carrera de Biología, Facultad de Estudios Superiores Iztacala Universidad Nacional Autónoma de Meéxico Tlalnepantla de Baz Mexico; ^2^ Posgrado en Ciencias Biológicas Universidad Nacional Autónoma de México Ciudad de México Mexico; ^3^ Departamento de Ecología y Recursos Naturales, Facultad de Ciencias Universidad Nacional Autónoma de México, Ciudad Universitaria Ciudad de México Mexico; ^4^ Museo de Zoología, Facultad de Ciencias Universidad Nacional Autónoma de México, Ciudad Universitaria Ciudad de México Mexico

**Keywords:** comparative analysis, evolutionary patterns, lizards, phenotype evolution, Phrynosomatidae

## Abstract

The evolution of phenotype has historically been studied by classifying traits into categories, as traits within each category often exhibit close associations. However, these categories are not independent of one another. Thus, the phenotype may function as an integrated set of traits rather than as isolated units. In this study, we employed various phylogenetic comparative methods to explore evolutionary correlations among traits, estimate and compare phylogenetic signals, and evaluate evolutionary models to assess the validity of the historical categorization of phenotypic traits in spiny lizards of the genus *Sceloporus*. We categorized these traits as either morphological or life‐history traits, including eight morphological traits and seven life‐history traits, such as trunk length, head width, snout‐vent length, clutch size, hatchling length, and size at maturity. Our analyses revealed covariation in the evolution of traits both within and across categories. Differences in phylogenetic signals between categories were also observed, though these results should be interpreted with caution. Additionally, the evolutionary models varied between categories. Our findings suggest that estimates of phylogenetic signals and covariation between morphological and life‐history traits are independent of historically assigned categories. Therefore, this supports the notion that traits should be compared and analyzed in an integrated manner, regardless of their category. We discuss how evolutionary mechanisms, such as fecundity selection, may influence traits across different categories (e.g., trunk length, hatchling length, and clutch size), challenging the appropriateness of traditional phenotypic categorization in evolutionary studies of *Sceloporus*.

## Graphical abstract

We investigated the evolutionary relationships between morphological and life‐history traits in *Sceloporus* lizards using phylogenetic comparative methods. Our results show covariation in trait evolution both within and across traditional trait categories, suggesting that phenotypic traits function as an integrated set rather than isolated units. These findings challenge the historical categorization of traits and highlight the importance of analyzing phenotype in a more holistic manner.

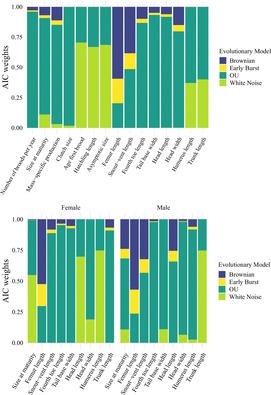

The concept of “trait” as a predictor (proxy) of organism performance (Darwin 1859) is fundamental in evolutionary biology. Historically, the traits composing the phenotype have been classified into categories such as physiological, behavioral, morphological, or life‐history traits. This classification facilitates the compartmentalization of phenotypic evolution studies, allowing for a deeper understanding of how traits within a specific category affect fitness both individually and collectively. For instance, morphological traits are defined by the intrinsic relationship between their form and function (Losos 1990), whereas life‐history traits are directly linked to key aspects of fitness, being associated with reproduction (Roff 2002).

In reptiles, studies of morphological and life‐history traits often focus on specific traits. The most studied morphological traits include head dimensions (width, length, and height), limb and tail lengths (Losos 1990; Molina‐Borja and Rodríguez‐Domínguez 2004; Goodman et al. 2008; Pincheira‐Donoso et al. 2009; Mahler et al. 2010; Hertz et al. 2013; Oufiero and Gartner 2014). For life‐history traits, the most studied include female size and age at sexual maturity, clutch or litter size and frequency, neonate size, and longevity (Stearns 1984; Bauwens and Diaz‐Uriarte 1997; Molina‐Borja and Rodríguez‐Domínguez 2004; Niewiarowski et al. 2004; Mesquita et al. 2016; Zúñiga‐Vega et al. 2016, 2017). Body size (defined as snout‐vent length) is a special case, as it has been considered both a morphological trait (Losos 1990; Molina‐Borja and Rodríguez‐Domínguez 2004; Goodman et al. 2008; Pincheira‐Donoso et al. 2009; Mahler et al. 2010; Hertz et al. 2013; Oufiero and Gartner 2014) and a life‐history trait (Stearns 1984; Bauwens and Diaz‐Uriarte 1997; Niewiarowski et al. 2004; Zúñiga‐Vega et al. 2016, 2017), reflecting its broad influence on fitness and the complexity of categorizing phenotypic traits.

The categorical study of phenotype evolution is justified, as traits within a category often exhibit close association (Hallgrímsson and Hall 2011). For example, body size is related to many other morphological traits, while maturation, litter size, and survival are also interconnected (McCoy et al. 2006; Mesquita et al. 2016). Additionally, in some cases, traits may exhibit a negative relationship (trade‐off), meaning that the optimal development of one trait imposes costs on another. For instance, in many species, an increase in offspring size leads to a reduction in their number (Bauwens and Diaz‐Uriarte 1997; Ljungström et al. 2016). Trait categorization provides a structured approach for studying phenotype evolution and acknowledges that traits within categories frequently interact to enhance fitness. Beyond its practical utility, this classification also has conceptual roots: it has been proposed that traits more directly related to fitness—such as life‐history traits—tend to exhibit lower heritability than morphological traits, due to stronger selective pressures (Mousseau and Roff 1987). However, the different categories that compose the phenotype are not independent; certain traits from one category may interact with those from another. For instance, interlimb length (trunk length) is positively associated with litter size (Olsson et al. 2002; Kratochvíl et al. 2003; Scharf and Meiri 2013). Consequently, since organisms experience selective forces as whole entities, phenotypes should be studied in an integrative manner, where specific traits are considered components of a broader phenotype rather than isolated units.

Although selective pressures can act on the entire phenotype, it is essential to consider that trait evolution may experience delays, as evolutionary pressures vary in intensity and duration over time (Grant and Grant 2007). For example, at the onset of an adaptive radiation or following a mass extinction, morphological disparity tends to increase due to the ecological opportunities that influence the rate of diversification (Erwin 2007; Gavrilets and Losos 2009; Mahler et al. 2010). Additionally, habitat‐specific challenges can drive morphological specialization, as evidenced by adaptive changes in body structure (Wiens et al. 2006; Goodman et al. 2008). Furthermore, evolutionary trade‐offs may constrain traits, as in the case where larger offspring size leads to smaller litter sizes (Bauwens and Diaz‐Uriarte 1997; Ljungström et al. 2016). Finally, key innovations such as viviparity can influence the evolution of other traits due to the reproductive costs involved (Oufiero and Gartner 2014; Zúñiga‐Vega et al. 2016). These variations in the intensity and persistence of selective pressures, along with their effects on traits, highlight the complexity of the phenotype. Therefore, evolutionary studies may be more effective when approached from an integrative perspective, regardless of historical trait categorization.

The phylogenetic signal, evolutionary patterns, and evolutionary trade‐offs have been estimated in different trait categories such as physiological (Baeckens et al. 2018; Mangiacotti et al. 2021), environmental (Baeckens et al. 2015; Winchell et al. 2020), behavioral (Baeckens et al. 2017), morphological (Gabelaia et al. 2017; Jara et al. 2018), and life‐history (Mesquita et al. 2016; Zúñiga‐Vega et al. 2017). However, this understanding may be influenced by the historical categorization of phenotypic traits. In this study, we used different comparative methods to evaluate the validity of the historical categorization of traits in the evolutionary study of phenotype, focusing specifically on traits traditionally classified as morphological and life‐history of the American spiny lizard's genus *Sceloporus*. This genus is the most diverse within the family Phrynosomatidae, with 117 species distributed across North and Central America, from southern Canada to Costa Rica. These lizards exhibit both viviparous and oviparous reproductive strategies and inhabit diverse environments (Uetz et al. 2024). We focused on: (1) exploring the multivariate evolution among traits within the morphological and life‐history categories, (2) estimating and comparing the phylogenetic signal between categories, and (3) fitting different evolutionary models for these traits. We considered that if the historical categorization of phenotypic traits is appropriate for their evolutionary study, traits from different categories should not covary in their evolution, and phylogenetic signal and evolutionary models should differ noticeably between morphological and life‐history traits. Specifically, we anticipate a lower intensity of phylogenetic signal and random evolution in morphological traits, as they are often assumed to respond rapidly to environmental pressures, leading to the perception that these traits are poorly conserved in phylogeny (Wiens et al. 2006; Goodman et al. 2008; Mahler et al. 2010).

## 1 Materials and Methods

We compiled a list of the 117 species of *Sceloporus* recognized until 2024, excluding subspecies (Uetz et al. 2024). Using this list, including as many species as possible with available morphological and life‐history trait data. We measured 11 morphological traits of 78 species using specimens housed at the Museo de Zoología “Alfonso L. Herrera”, Facultad de Ciencias, Univesidad Nacional Autónoma de México, UNAM (MZFC‐H). The morphological traits we measured were head width (HW; maximum distance between the supraocular scales on both sides of the head), head length (HL; distance from the tip of the snout to the posterior edge of the tympanum), trunk length (TL; distance from the axilla to the groin), right humerus length (RHuL; distance from the right axilla to the right elbow), left humerus length (LHuL; distance from the left axilla to the left elbow), right femur length (RFL; distance from the right groin to the right knee), left femur length (LFL; distance from the left groin to the left knee), right fourth toe length (4RT; distance from the base of the claw to the pad of the right hind limb), left fourth toe length (4LT; distance from the base of the claw to the pad of the left hind limb), tail base width (TBW; maximum width immediately posterior to the cloacal opening), and snout‐vent length (SVL; distance from the tip of the snout to the cloacal opening; Data S1). All measurements were taken by a single author using a digital caliper with an accuracy of 0.01 mm (Mitutoyo Model 500‐196‐30). Additionally, we obtained data for seven life‐history traits primarily through an extensive literature review. The life‐history traits included size at maturity (SM), asymptotic size (AS; maximum reported SVL), clutch size (CS), hatchling length (HL), mass‐specific production (MP), number of broods per year (YB), age at first brood (AFB; Data S1). The subsequent analyses were performed using the log‐transformed data.

All morphological trait measurements were taken from adult organisms. We differentiated juveniles from adults primarily through direct observation of enlarged follicles and testes, supplemented with information on size at maturity. Given the sexual dimorphism present in *Sceloporus* (see Jiménez‐Arcos et al. 2017), we ensured a balanced ratio of male and female specimens for each species, with a sample size ranging from three to 20 specimens per sex. Species averages were calculated using all specimens (Data S1). Except for SM, which was recorded separately for each sex, all life‐history traits were analyzed at the species level. Since no significant differences were observed between the left and right paired morphological traits (RHuL—LhuL: AIC_null model_ = 16450.1, AIC_group model_ = 16452.18, ⊗AIC = 2.08; RFL—LFL: AIC_null model_ = 18996.17, AIC_group model_ = 18998.42, ⊗AIC = 2.25; 4RT—4LT: AIC_null model_ = 18051.1, AIC_group model_ = 18053.78, ⊗AIC = 2.68), we calculated the average of both traits and used it as a single trait (HuL, FL, 4T).

Considering that some populations might prove to be distinct taxa in future taxonomic work, we limited our measurements to specimens from a single population, preferably the type locality or the nearest available one. Regarding life‐history traits, we compiled the maximum amount of available information. Unfortunately, we were unable to obtain complete data on every trait for all species, either through direct measurements or a literature review. Consequently, the sample size for each analysis varied between 34 and 95 species. Although the dataset compiled here does not include information from all *Sceloporus* species, it is one of the most comprehensive in terms of the number of species and quantity of traits within a genus of lizards. We selected the traits analyzed in this study based on their frequency in previous comparative studies (Bauwens and Diaz‐Uriarte 1997; Molina‐Borja and Rodríguez‐Domínguez 2004; Mahler et al. 2010; Hertz et al. 2013; Oufiero and Gartner 2014; Mesquita et al. 2016).

We explored the multivariate evolution of phenotypic traits using the “*mvMORPH”* package (Clavel et al. 2015) in R (R Core Team 2024) to fit two multivariate models of continuous trait evolution: the Brownian motion (BM) model and the Ornstein‐Uhlenbeck (OU) model, implemented via the *mvBM* and *mvOU* functions, respectively. These models allow us to describe the correlated evolution of traits without reducing their dimensionality or assuming a fixed response‐predictor variable structure, unlike other multivariate methods such as phylogenetic principal components analysis (phyl PCA; Revell 2012) or generalized linear mixed models (GLMM; Hadfield 2010). The BM model represents trait evolution as a random process at a constant rate (Blomberg et al. 2003), whereas the OU model incorporates a stabilizing force that drives traits towards an adaptive optimum (Butler and King 2004). Both multivariate models allow us to evaluate whether morphological and life‐history trait categories exhibit correlated evolution.

We performed model selection based on the Akaike information criterion (AIC; Burnham and Anderson 2002). This criterion favors the model that achieves the best balance between its complexity and its goodness‐of‐fit. The lowest AIC value indicates the best‐fitting model, and a difference in AIC values greater than two units between the evaluated models (⊗AIC > 2) suggests substantial differences in their fit to the data (Burnham and Anderson 2004).

We estimated the phylogenetic signal of each trait using two different methods, λ (Pagel 1999) and *K* (Blomberg et al. 2003). Both calculations were performed using the *phylosig* function in the *“phytools”* package (Revell 2012) in R. These two measures of phylogenetic signal utilize phenotypic data and the phylogenetic variance–covariance matrix as inputs, but they are calculated differently. λ quantifies the degree to which the phylogenetic variance–covariance matrix explains the phenotypic data by rescaling the tree's branch lengths to maximize the fit. *K* compares the observed mean squared error of phenotypic data across the tree to the mean squared error expected under a BM model, assessing the proportional similarity of traits among related species (Münkemüller et al. 2012). Both measures can range from zero to values above one, where λ = 0 and *K* = 0 indicate that the trait has evolved independently of the phylogeny, meaning there is no phylogenetic signal, and hence close relatives are no more similar on average than distant relatives. λ = 1 and *K* = 1 indicate that the trait has evolved according to a Brownian motion model, meaning there is strong phylogenetic signal, and hence close relatives are more similar on average than distant relatives. Values of λ and *K* greater than one indicate that close relatives are more similar than expected under a BM model of trait evolution. To assess whether λ was significantly different from zero, we employed a likelihood ratio test comparing the model with the observed λ value to a model where λ is fixed at zero. For *K*, we used a randomization test that permutes *n* times the trait values across the phylogeny, calculates *n* new *K* values, and compares the observed *K* to the distribution of obtained *K* values (Münkemüller et al. 2012).

To quantify the uncertainty in the phylogenetic signal associated with the phylogenetic reconstruction, we randomly selected a tree from the posterior sample of the phylogenetic tree distribution (available in Domínguez‐Guerrero et al. 2022). Using this tree, we calculated the phylogenetic signal of the morphological and life‐history traits. Subsequently, we performed a bootstrap analysis by resampling 10,000 times the differences between the phylogenetic signal values obtained from the Maximum Clade Credibility Tree and those from the randomly selected tree from the posterior sample. This bootstrap analysis estimates the variability of these differences, providing a robust measure of uncertainty. Finally, we reported the distribution of the mean differences, the mean of those differences, and the 95% confidence intervals.

We tested for potential differences in the phylogenetic signal between both trait categories by constructing two linear models. The first was an intercept‐only (null) model, in which the phylogenetic signal values of both groups were treated as the response variable. In the second model, the phylogenetic signal values were the response variable, and the categories (morphological and life‐history traits) were the predictor variables. We compared the fit of these models using AIC and implemented them using the base R package.

Phylogenetic signal is defined as the non‐independence of trait values among species due to their shared evolutionary history (Blomberg et al. 2003; Revell et al. 2008). Specifically, it quantifies the relationship between phylogeny and phenotypic similarity among species, serving as a statistical descriptor rather than a causal explanation for the resemblance between related species (Revell et al. 2008; Losos 2008). To better understand the mechanisms underlying the evolution of morphological and life‐history traits, we evaluated and compared the fit of four evolutionary models of trait evolution using the *fitContinuous* function from “*geiger*” package (Harmon et al. 2008) in R. The models fitted were: (1) White Noise (WN), a null model assuming no phylogenetic structure (Pennell et al. 2014); (2) Brownian Motion (BM), which represents random evolution with constant variance over time (Blomberg et al. 2003); (3) Early Burst (EB), describing a decelerating or accelerating rate of evolution through time (Harmon et al. 2010); and (4) Ornstein‐Uhlenbeck (OU), incorporating stabilizing selection towards an adaptative optimum (Butler and King 2004). We again compared the fit of these models using AIC.

For these analyses, we used the Maximum Clade Credibility Tree provided by Domínguez‐Guerrero et al. (2022), as it is the most comprehensive tree available for phrynosomatid lizards. We pruned the tree using the *keep.tip* function from the “*ape*” package (Paradis 2012) in R to remove species for which we had no data. This pruning was adjusted for each sex and trait due to differences in data availability.

## 2 Results

**FIGURE 1:**
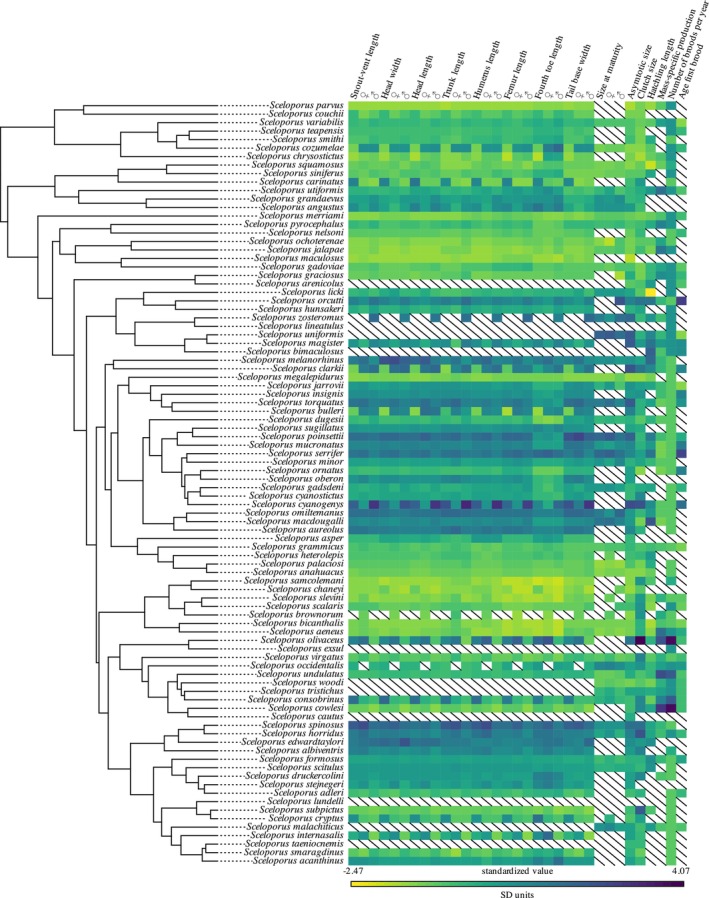
Phylogeny of the genus *Sceloporus* including the species analyzed in this study. Standardized trait values are mapped onto the tips of the tree, where colors represent the intensity of standardized values for each phenotypic trait.

We obtained data from both sexes in 71 out of the 78 species of *Sceloporus*. In three species (*S. brownorum, S* *. olivaceus* , *S* *. squamosus* ), we measured only female specimens, while in four species (*S. angustus, S* *. magister* , *S* *. occidentalis* , *S. zosteromus*), we measured only male specimens. The amount of available life‐history traits data was heterogeneous. Regarding the sample size per trait, we obtained data for size at maturity in 52 species, for asymptotic size in 93 species, for clutch size in 82 species, for hatchling length in 61 species, for mass‐specific production in 45 species, for number of broods per year in 79 species, and for age at first brood in 40 species (Figure 1).

The selection of multivariate models for trait evolution showed that the OU model had the lowest AIC value (2230.24) compared to the BM model (2344.44, ΔAIC = 113.69). This indicates that the model incorporating a stabilizing force towards an adaptive optimum provides a better fit to the data compared to the model of random evolution.

**FIGURE 2:**
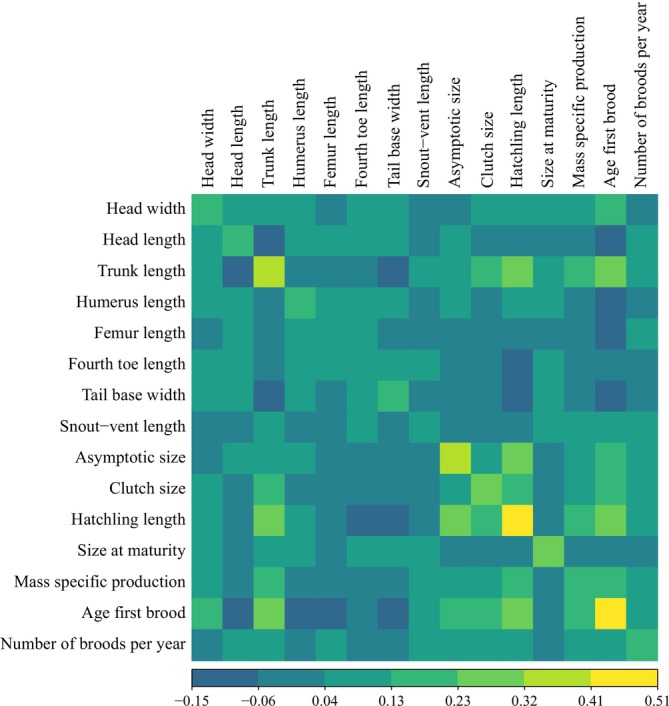
Variance–covariance matrix estimated by the multivariate Ornstein‐Uhlenbeck (OU) model for morphological and life‐history traits of *Sceloporus* lizards. Colors indicate the magnitude and direction of standardized covariances between trait pairs.

The phylogenetic variance–covariance matrix derived from the OU model reflected covariation in the evolution of traits both within and between categories. Among morphological traits, near‐zero covariation (< 0.1) was observed between head length, tail base width, and SVL with head width; between femur length and humerus length; and between trunk length and both humerus length and fourth toe length. For life‐history traits, near‐zero covariation was notable between asymptotic size and mass‐specific production; between size at maturity and clutch size; as well as between size at maturity and both number of broods per year and age at first brood. This suggests that, overall, life‐history traits exhibit less near‐zero covariation compared to morphological traits (Figure 2).

The phylogenetic variance–covariance matrix showed positive covariation (> 0.1) between traits from different categories, specifically between clutch size, hatchling length, mass‐specific production, and age at first brood with trunk length (Figure 2). These were the only positive covariations between traits from different categories. This pattern suggests that the categories of traits do not evolve as entirely independent blocks and that complex interactions exist in trait evolution.

We observed phylogenetic signal in 12 out of the 15 traits analyzed, using both λ and *K* (Table 1). Except for female SLV, where λ had a substantially lower value (< 0.01, *p* = 1) compared to *K* (0.46, *p* = 0.05), in all other traits the magnitude of phylogenetic signal was similar when evaluated with both methods (Table 1). The traits that did not show significant phylogenetic signal were asymptotic size (λ = 0.19, *p* = 0.07; *K* = 0.4, *p* = 0.22), hatchling length (λ < 0.01, *p* = 1; *K* = 0.31, *p* = 0.46), and age first brood (λ = 0.42, *p* = 0.15; *K* = 0.4, *p* = 0.22; Table 1).

**TABLE 1:** Phylogenetic signal and sample size (*n*) for eight morphological traits and seven life‐history traits of lizard species of the genus *Sceloporus*. The values of λ and *K* are provided with their respective *p*‐values. In all tests, significance values were set at 0.05.

Trait	Species					Male					Female				
	*n*	λ	*p*	*K*	*p*	*n*	λ	*p*	*K*	*p*	*n*	λ	*p*	*K*	*p*
Morphological															
Head width (HW)	78	0.83	< 0.01	0.47	< 0.01	75	0.66	< 0.01	0.44	< 0.01	74	0.67	< 0.01	0.3	0.11
Head length (HL)	78	0.79	< 0.01	0.48	< 0.01	75	0.82	< 0.01	0.49	< 0.01	74	0.43	< 0.01	0.25	0.34
Trunk length (TL)	78	0.33	< 0.01	0.34	0.07	75	0.19	0.4	0.3	0.18	74	0.71	< 0.01	0.48	< 0.01
Humerus length (HuL)	78	0.31	0.28	0.41	< 0.01	75	0.89	0.03	0.46	< 0.01	74	0.07	0.82	0.24	0.5
Femur length (FL)	78	0.94	< 0.01	1.04	< 0.01	75	0.94	< 0.01	0.82	< 0.01	74	0.89	< 0.01	0.84	< 0.01
Fourth toe length (4 T)	78	0.78	< 0.01	0.8	< 0.01	75	0.67	< 0.01	0.64	< 0.01	74	0.72	< 0.01	0.66	< 0.01
Tail base width (TBW)	78	0.72	< 0.01	0.59	< 0.01	75	0.43	< 0.01	0.42	< 0.01	74	0.74	< 0.01	0.59	< 0.01
Snout‐vent length (SVL)	78	0.9	< 0.01	0.84	< 0.01	75	0.83	< 0.01	0.67	< 0.01	74	0.82	< 0.01	0.69	< 0.01
Life‐history															
Size at maturity (SM)	52	0.8	< 0.01	0.57	< 0.01	47	0.81	0.01	0.67	< 0.01	47	< 0.01	1	0.46	0.05
Asymptotic size (AS)	93	0.19	0.07	0.27	0.18										
Clutch size (CS)	82	0.58	< 0.01	0.41	< 0.01										
Hatchling length (HL)	61	< 0.01	1	0.31	0.46										
Mass‐specific production (MP)	45	0.63	< 0.01	0.53	0.05										
Number of broods per year (YB)	79	0.78	< 0.01	0.6	< 0.01										
Age first brood (AFB)	40	0.42	0.15	0.4	0.22										

To evaluate potential differences in the phylogenetic signal values obtained using both methods (λ and K), we compared two models. The first was a null model (intercept‐only), which assumed no differences between the phylogenetic signal values generated by the two methods. The second model included the method as a predictor variable, allowing us to evaluate whether the signal values differ depending on the method employed. The intercept‐only model had the lowest AIC value (4.09 compared to 5.52) However, there is insufficient evidence to discard the model including the method as a predictor variable (ΔAIC = 1.42). Akaike weights indicate that the null model is more supported (AICwnull = 0.67) relative to the predictor variable model (AICw = 0.33), suggesting no significant differences between the methods used. Given the consistency of results between λ and *K*, and since *K* can also detect cases where traits exhibit a level of similarity greater than expected under a BM model, we focused on the results using *K*.

**FIGURE 3:**
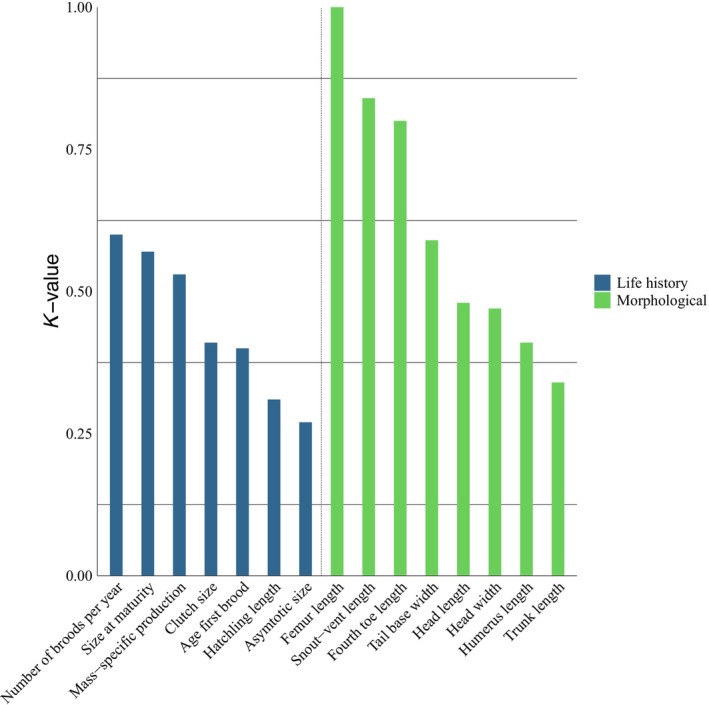
Phylogenetic signal of morphological and life‐history traits of *Sceloporus* lizards quantified by means of the parameter *K*.

The values of *K* for morphological traits (without considering differences between sexes) ranged from 0.47 to 1 (Figure 3). When analyzing the phylogenetic signal of these traits by sex, we observed pronounced differences in trunk length (higher in females) and tail base width (higher in males). Specifically, TL in males had the lowest phylogenetic signal (*K* = 0.3). Additionally, we recorded a non‐significant phylogenetic signal in head width, head length, and humerus length in females (Table 1).

In life‐history traits, *K* values (without considering differences between sexes) showed moderate variation, ranging from 0.27 to 0.6 (Figure 3). Furthermore, moderate variation in phylogenetic signal between sexes was observed in size at maturity, with males exhibiting a higher value. Finally, non‐significant phylogenetic signals were found in asymptotic size, hatchling length, and age at first brood (Table 1).

**FIGURE 4:**
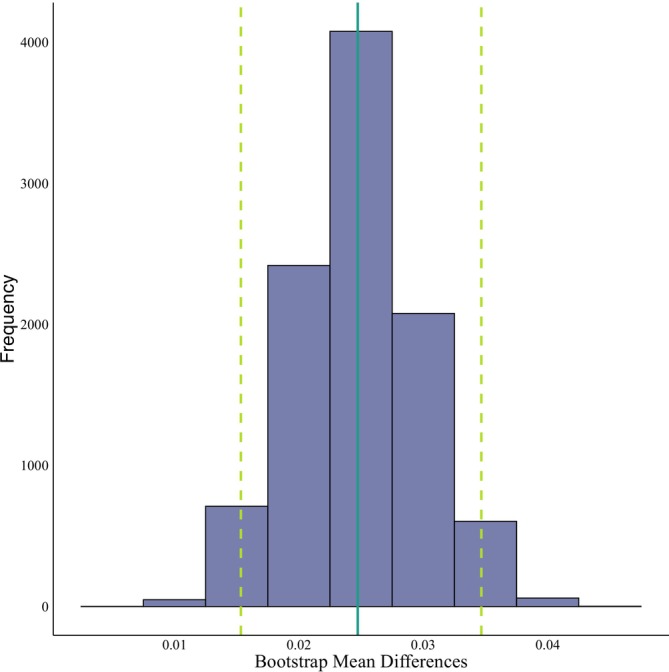
Distribution of mean differences obtained through the bootstrap analysis for phylogenetic signal. The solid vertical line indicates the mean of the resampled mean differences. Dotted lines delimit the confidence interval.

The bootstrap analysis revealed that the mean of the differences between the resampled means (0.0247) was close to the originally calculated value (0.0246). Additionally, the confidence interval was relatively narrow (0.0153–0.0346), indicating stable results and low uncertainty associated with phylogenetic reconstruction in the calculation of phylogenetic signal (Figure 4).

The model comparison did not provide strong evidence to rule out differences in phylogenetic signal values estimated for morphological traits and life‐history traits. The model incorporating trait categories as a predictor variable and phylogenetic signal values as the response variable had a lower AIC value (−2.76) compared to the null model (−1.62). Although there is not enough evidence to strongly prefer the model describing differences in estimated phylogenetic signal values between the two categories over the model assuming homogeneity (ΔAIC = 1.14), Akaike weights suggest greater relative support for the former (AICw = 0.64 versus AICwnull = 0.36) indicating that it provides a slightly better fit to the data. The observed differences in the mean phylogenetic signal values between morphological traits (mean = 0.61, SD = 0.23) and life‐history traits (mean = 0.44, SD = 0.12) are consistent with this model; however, this should be interpreted cautiously due to the relatively small ΔAIC.

**FIGURE 5:**
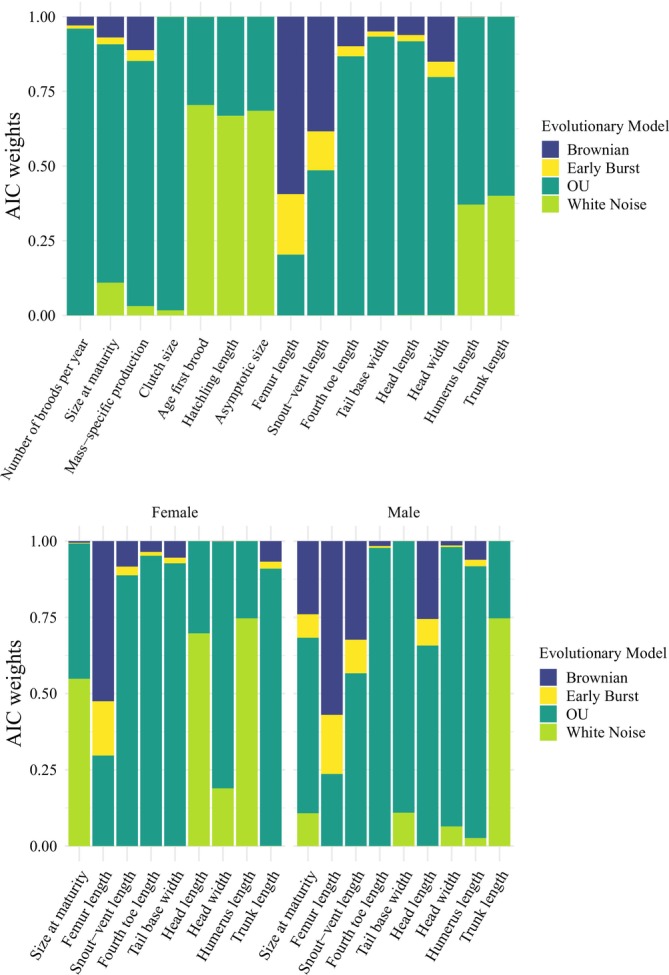
Akaike weights (AICw) for the four evolutionary models evaluated for each trait. The models included are Brownian Motion (BM), Ornstein‐Uhlenbeck (OU), Early Burst (EB), and White Noise (WN). The bars' colors reflect the contribution of each model in terms of its fit to the data. Upper panel shows models evaluated at the species level, while the bottom panel shows models evaluated by sex.

The evolutionary model fitting of morphological and life‐history traits per species showed that the OU model provided the best fit for most traits (Table 2). Additionally, the WN model was the best fit for three traits: age at first brood, hatchling length, and asymptotic size (Figure 5). The BM model best fit femur length, while the OU model fit the remaining traits best. The model fitting also revealed differences between sexes (Figure 5). For females, size at maturity, head length, and humerus length were best fit by the WN model, while for males, the OU model provided the best fit for these traits (Table 2). For trunk length, the best‐fitting model was WN for males and OU for females (Figure 5). For the remaining traits, the OU model was the best fit for both sexes, except for femur length, where the BM model performed best. No trait had EB as the best‐fitting model (Table 2).

**TABLE 2:** Evolutionary model selection for different morphological and life‐history traits at two levels (species and sex) in lizards of the genus *Sceloporus*. Four evolutionary models were evaluated for each trait: Brownian Motion (BM), Ornstein‐Uhlenbeck (OU), Early Burst (EB), and White Noise (WN). Reported values include the corrected Akaike Information Criterion for small samples (AICc), the difference in AICc relative to the best model (ΔAICc), and model weight (AICw).

Trait	Species				Male				Female			
Morfological												
Head width	Model	AICc	ΔAICc	AICw	Model	AICc	ΔAICc	AICw	Model	AICc	ΔAICc	AICw
	*OU*	−257.58	0	0.79	*OU*	−224.51	0	0.91	*OU*	−227.97	0	0.8
	*BM*	−254.15	3.43	0.25	*WN*	−219.19	5.32	0.06	*WN*	−225.06	2.91	0.18
	*EB*	−251.99	5.59	0.05	*BM*	−216.22	8.29	0.01	*BM*	−214.34	13.63	< 0.01
	*WN*	−242.92	14.66	< 0.01	*EB*	−214.06	10.45	< 0.01	*EB*	−212.18	15.79	< 0.01
Head length	*OU*	−289.47	0	0.91	*OU*	−272.53	0	0.65	*WN*	−253.43	0	0.69
	*BM*	−284.06	5.41	0.06	*BM*	−270.64	1.89	0.25	*OU*	−251.76	1.67	0.3
	*EB*	−281.9	7.57	0.02	*EB*	−268.48	4.05	0.08	*BM*	−222.26	31.17	< 0.01
	*WN*	−275.03	14.44	< 0.01	*WN*	−256.37	16.16	< 0.01	*EB*	−220.1	33.33	< 0.01
Trunk length	*OU*	−292.51	0	0.59	*WN*	−269.25	0	0.74	*OU*	−277.97	0	0.9
	*WN*	−291.7	0.81	0.4	*OU*	−267.09	2.16	0.25	*BM*	−272.77	5.2	0.06
	*BM*	−271.84	20.67	< 0.01	*BM*	−243.51	15.74	< 0.01	*EB*	−270.6	7.37	0.02
	*EB*	−269.68	22.83	< 0.01	*EB*	−241.34	27.91	< 0.01	*WN*	−262.28	15.69	< 0.01
Humerus length	*OU*	−249.91	0	0.62	*OU*	−247.43	0	0.89	*WN*	−218.51	0	0.74
	*WN*	−248.86	1.05	0.37	*BM*	−242.08	5.35	0.06	*OU*	−216.35	2.16	0.25
	*BM*	−237.99	11.92	< 0.01	*WN*	−240.35	7.08	0.02	*BM*	−181.86	26.65	< 0.01
	*EB*	−235.83	14.08	< 0.01	*EB*	−239.92	7.51	0.02	*EB*	−179.69	38.82	< 0.01
Femur length	*BM*	−236.68	0	0.59	*BM*	−229.91	0	0.57	*BM*	−212.97	0	0.52
	*OU*	−234.54	2.14	0.2	*OU*	−228.15	1.76	0.23	*OU*	−211.83	1.14	0.29
	*EB*	−234.52	2.16	0.2	*EB*	−227.75	2.16	0.19	*EB*	−210.8	2.17	0.17
	*WN*	−174.23	62.45	< 0.01	*WN*	−184.63	45.28	< 0.01	*WN*	−162.85	50.12	< 0.01
Fourth toe length	*OU*	−158.38	0	0.86	*OU*	−151.86	0	0.97	*OU*	−141.71	0	0.95
	*BM*	−154.04	4.34	0.09	*BM*	−143.67	8.19	0.01	*BM*	−135.14	6.57	0.03
	*EB*	−151.88	6.5	0.03	*EB*	−141.51	10.35	< 0.01	*EB*	−132.98	8.73	0.01
	*WN*	−125.75	32.63	< 0.01	*WN*	−130.01	21.85	< 0.01	*WN*	−114.3	27.41	< 0.01
Tail base width	*OU*	−229.33	0	0.93	*OU*	−189.35	0	0.89	*OU*	−212.41	0	0.92
	*BM*	−223.47	5.86	0.04	*WN*	−185.15	4.2	0.1	*BM*	−206.74	5.67	0.05
	*EB*	−221.3	8.03	0.01	*BM*	−172.8	16.55	< 0.01	*EB*	−204.57	7.84	0.01
	*WN*	−206.25	23.08	< 0.01	*EB*	−170.63	18.72	< 0.01	*WN*	−184.73	27.68	< 0.01
Snout‐vent length	*OU*	−32.55	0	0.48	*OU*	−16.96	0	0.56	*OU*	−32.73	0	0.88
	*BM*	−32.08	0.47	0.38	*BM*	−15.84	1.12	0.32	*BM*	−28.01	4.72	0.08
	*EB*	−29.92	2.63	0.13	*EB*	−13.67	3.29	0.1	*EB*	−25.84	6.89	0.02
	*WN*	1.17	33.72	< 0.01	*WN*	12.76	29.72	< 0.01	*WN*	−6.75	25.98	< 0.01
Life‐history												
Size at maturity	*OU*	−50.77	0	0.79	*OU*	−41.66	0	0.57	*WN*	−49.82	0	0.54
	*WN*	−46.8	3.97	0.1	*BM*	−39.91	1.75	0.24	*OU*	−49.4	0.42	0.44
	*BM*	−45.9	4.87	0.02	*WN*	−38.3	3.36	0.1	*BM*	−40.71	9.11	< 0.01
	*EB*	−43.64	7.13	0.06	*EB*	−37.63	4.03	0.07	*EB*	−38.43	11.39	< 0.01
Asymptotic size	*WN*	−108.85	0	0.68								
	*OU*	−107.3	1.55	0.31								
	*BM*	−66.29	42.56	< 0.01								
	*EB*	−64.14	44.71	< 0.01								
Clutch size	*OU*	50.24	0	0.98								
	*WN*	58.37	8.13	0.01								
	*BM*	67.47	17.23	< 0.01								
	*EB*	69.63	19.39	< 0.01								
Hatchling length	*WN*	−68.82	0	0.66								
	*OU*	−67.42	1.4	0.33								
	*BM*	−43.84	24.98	< 0.01								
	*EB*	−41.62	27.2	< 0.01								
Mass‐specific production	*OU*	76.98	0	0.82								
	*BM*	80.96	3.98	0.11								
	*EB*	83.24	6.26	0.03								
	*WN*	83.54	6.56	0.03								
Yearly broods	*OU*	136.38	0	0.96								
	*BM*	143.34	6.96	0.02								
	*EB*	145.5	9.12	0.01								
	*WN*	164	27.62	< 0.01								
Age first brood	*WN*	282.62	0	0.7								
	*OU*	284.36	1.74	0.29								
	*BM*	297.46	14.84	< 0.01								
	*EB*	299.8	17.18	< 0.01								

## 3 Discussion

Our multivariate analysis of phenotypic trait evolution revealed covariation in the evolution of certain traits across both categories (Figure 2). This result contrasts with our hypothesis, which posited that if the historical categorization of phenotypes is appropriate for evolutionary studies, there should be no covariation between traits from different categories, and the phylogenetic signal and evolutionary models of traits from each category should differ. This finding suggests that the historical classification of traits as morphological or life‐history does not reflect the interrelationship between both types of traits and their evolution, at least for the studied group of lizards. Nonetheless, comparisons of phylogenetic signal suggest differences between the two categories, though this result should be interpreted cautiously. Additionally, we observed notable differences in the evolutionary models describing traits in each category (Figure 5). We interpret these results as mixed evidence regarding the integration of the phenotype in evolutionary analysis. While patterns of covariation between categories suggest complex interactions in phenotypic evolution, we did not find conclusive evidence to accept or reject the historical categorization of the phenotype. Therefore, we recommend that future studies prioritize analyzing underlying evolutionary mechanisms rather than focusing exclusively on predefined categories.

We anticipated weaker phylogenetic signal and random evolution in morphological traits, as these are often assumed to respond quickly to environmental pressures, leading to the perception that they are poorly conserved in phylogeny (Wiens et al. 2006; Goodman et al. 2008; Mahler et al. 2010). However, we observed notable variation in the phylogenetic signal estimated for morphological traits, consistent with previous studies on other lizard genera, albeit in traits from a different category (Zúñiga‐Vega et al. 2017). Although formal studies on the phylogenetic signal of general body shape traits in lizards are limited, those that include SVL report moderate (Baeckens et al. 2015; Zúñiga‐Vega et al. 2017) to high values (Mesquita et al. 2016; Mangiacotti et al. 2021), which aligns with our estimates of phylogenetic signal for SVL (Table 1). This variation suggests that, for traits related to body size and shape in *Sceloporus*, common ancestry may have a limited impact on trait variation (Blomberg et al. 2003; Losos 2008), contrasting with the null effect we initially anticipated.

Life‐history traits showed considerable and relatively homogeneous values of phylogenetic signal (Figure 3). When increasing the taxonomic category of analysis, such as considering superfamilies, the phylogenetic signal of life‐history traits is high and homogeneous (Mesquita et al. 2016). Although the phylogenetic signal of traits in taxonomic categories lower than species has not been formally calculated, extensive variation in life‐history traits among populations of the same species within *Sceloporus* has been reported. Specifically, traits such as size at maturity and age at maturity exhibit substantial variation among populations. In contrast, other traits such as clutch size and hatchling length show much less variation among populations (Ramírez‐Bautista et al. 2004; Niewiarowski et al. 2004; Pérez‐Mendoza and Zúñiga‐Vega 2014). This may suggest that shared ancestry could have a limited impact on the variation of life‐history traits among populations (Niewiarowski et al. 2004), resulting in homogeneous phylogenetic signal at larger levels of analysis and heterogeneous signals at lower levels. However, it is essential to consider that the taxonomic scale of the phylogeny used influences the calculation of phylogenetic signal (Losos 2008). If the phylogeny does not include different populations of the same species, the variation that may exist at lower taxonomic levels will be reduced to a single value since the calculation of phylogenetic signal is limited to one observation per terminal branch of the phylogeny (Losos 2008). Therefore, we recommend not extrapolating the phylogenetic signal results obtained at one taxonomic level to another and paying close attention to the taxonomic level when comparing results.

Our results indicate a difference in the intensity of phylogenetic signal between morphological and life‐history traits in *Sceloporus*, with the latter showing a lower magnitude overall (Figure 3). Variation in the magnitude of phylogenetic signal can generally be observed across different trait categories that constitute the phenotype (Mesquita et al. 2016; Kamilar and Cooper 2013). However, assuming the potential phylogenetic signal of a trait based on its categorization a priori can be misleading, as our results for *Sceloporus* demonstrate. This highlights the need for caution when classifying traits according to preconceived expectations about their phylogenetic signal, as doing so may obscure meaningful evolutionary patterns (Kamilar and Cooper 2013).

Contrary to our predictions, most morphological traits evolved according to an OU model (Figure 5), indicating the action of stabilizing selection towards an adaptive optimum (Butler and King 2004). Three main mechanisms of morphological evolution can be recognized: sexual selection, fecundity selection, and habitat use (Olsson et al. 2002; Wiens et al. 2006; Cox et al. 2003; Goodman et al. 2008; Scharf and Meiri 2013). Previous studies have suggested evidence of sexual selection in *Sceloporus*; for example, most species exhibit sexual size dimorphism biased towards males, related to mating and agonistic encounters (Cox et al. 2003; Gienger and Beck 2007; Jiménez‐Arcos et al. 2017). However, the differences in the evolutionary model fit for SVL between sexes could suggest that other pressures drive changes in this trait in *Sceloporus*. This is reflected in the AIC difference between the OU and BM models (Table 2), which shows similar fits in males, suggesting greater complexity in the evolutionary processes affecting this sex.

A positive correlation between SVL and fecundity has been reported in female Sceloporus (Jiménez‐Arcos et al. 2017), which may reflect the action of fecundity selection, as proposed in other lizard taxa (Olsson et al. 2002). This is consistent with our results. However, SVL encompasses traits such as head and trunk length, which typically differ between sexes in several lizards (Olsson et al. 2002; Cox et al. 2003; Scharf and Meiri 2013). The intersexual differences in the evolutionary model fit for trunk and head length (Figure 5) and the negative covariation in their evolution (Figure 2) are consistent with previously reported sexual dimorphism in these traits (Kratochvíl et al. 2003; Scharf and Meiri 2013; Jiménez‐Arcos et al. 2017). While the OU fit for head length in males suggests the influence of sexual selection, potentially related to agonistic encounters or reproductive behaviors (Molina‐Borja et al. 1998; Gienger and Beck 2007), variation in this trait among females may also reflect the action of natural selection through trophic niche divergence (Schoener 1977; Shine 1989). Conversely, trunk length in males does not appear to be influenced by specific selective pressures, whereas in females, its OU fit may be consistent with the action of fecundity selection, a pattern documented in other lizard taxa (Olsson et al. 2002; Scharf and Meiri 2013).

Additionally, this pattern suggests that trunk length in females is more closely associated with fecundity selection than SVL (Scharf and Meiri 2013). While previous studies have shown that SVL is directly related to fecundity selection, as this pressure can influence female body size to accommodate more offspring (Cox et al. 2003; Jiménez‐Arcos et al. 2017), it is important to note that SVL includes other body parts, such as head length, which may be influenced by other selective pressures (Kratochvíl and Frynta 2002; Scharf and Meiri 2013; Brandt et al. 2016). In contrast, trunk length specifically reflects the body region where offspring are stored, making it a more direct indicator of fecundity selection (Olsson et al. 2002; Kratochvíl et al. 2003; Scharf and Meiri 2013).

In addition to the intersexual differences in trunk length, we observed that clutch size and hatchling length in *Sceloporus* show covariation in their evolution (Figure 2). This result supports the idea of fecundity selection affecting traits related to reproduction (Olsson et al. 2002; Cox et al. 2003; Scharf and Meiri 2013) and is consistent with a constraint due to a trade‐off between the number and size of offspring (Zúñiga‐Vega et al. 2017). As mentioned earlier, fecundity selection influences the number of offspring produced by females (Olsson et al. 2002; Cox et al. 2003; Scharf and Meiri 2013; Jiménez‐Arcos et al. 2017). It has been shown that when large offspring are produced, smaller litters result, and vice versa (Uller and Olsson 2005; Zúñiga‐Vega et al. 2017). Furthermore, this pattern is supported by the similar phylogenetic signal estimated for these traits, a characteristic often seen in correlated traits (Kamilar and Cooper 2013). This suggests that various traits, regardless of their category, may simultaneously evolve due to selective pressures acting on one or more traits.

Other traits that showed covariation with trunk length in their evolution were age at first brood and mass‐specific production (Figure 2). This could be attributed to their potential link with reproductive effort. Reproductive effort is influenced by the fecundity cost, determined by fecundity rate, reproductive frequency, and survival probability (Shine and Schwarzkopf 1992). Fecundity rate may increase or decrease with trunk length, reproductive frequency is linked to mass‐specific production, and survival is proportional to age at first brood (Shine and Schwarzkopf 1992; Shine and Charnov 1992). However, in reptiles, the optimal allocation of resources between growth, somatic maintenance, and reproduction may not be determined by a single selection mechanism (Shine and Schwarzkopf 1992). The lack of significant phylogenetic signal in age at first brood and mass‐specific production, along with the evolutionary model fit showing that these traits are not influenced by specific selective pressures, despite their correlation with other reproductive effort‐related traits, suggests that the mechanism affecting these traits may not be operating uniformly. This could indicate that other factors, such as local environmental conditions, exert greater influence on the evolution of age at first brood and mass‐specific production.

The historical division of the phenotype into categories—such as morphological, life‐history, physiological, and behavioral traits—is a longstanding conceptual proposal in evolutionary biology. One rationale behind this classification is the expectation that traits closely tied to fitness—such as life‐history traits—tend to exhibit lower heritability than morphological traits (Mousseau and Roff 1987). Additionally, it has been proposed that phenotypic correlations, particularly among morphological traits, can approximate genetic correlations, supporting their use in evolutionary studies when genetic data are unavailable (Cheverud 1988). Although our study focuses on macroevolutionary patterns, these perspectives highlight that trait categorization has historically reflected not only functional differences but also assumptions about genetic architecture and evolvability. While our results do not directly test these assumptions, the observed covariation and differences in evolutionary models suggest that such categorical distinctions may obscure rather than clarify evolutionary dynamics in *Sceloporus*.

In conclusion, our results indicate that estimates of phylogenetic signal and the covariation between morphological and life‐history traits are independent of the categories historically associated with them. This reinforces the idea that traits can be compared and analyzed integrally, regardless of their category, provided that an evolutionary mechanism (e.g., fecundity selection) is considered. Moreover, it is possible that this pattern is consistent with other traits not analyzed in this study. While phenotypic categories are useful as an initial framework, the evolution of traits should be examined through the lens of specific evolutionary mechanisms. Despite recommending this perspective for studying phenotype evolution, we acknowledge that an integrative approach remains a valuable tool and should not be dismissed. For instance, our results revealed covariation in the evolution of asymptotic size and age at first brood, which also shared the same evolutionary model and lack of significant phylogenetic signal. This finding could guide further research on reproductive effort. Similarly, the negative covariation observed between trunk length and head length, along with differences in phylogenetic signal and evolutionary models, could provide insights into how mechanisms such as fecundity selection and sexual selection interact in morphological evolution. However, it is important to note that this study represents an initial approximation, and further investigations involving additional interactions among phenotypic traits, such as habitat‐specific challenges, are necessary for a deeper understanding of phenotypic evolution in *Sceloporus*.

## Author Contributions

**Isaac Emmanuell Diaz‐Ortega:** conceptualization (lead), formal analysis (lead), investigation (lead), methodology (lead), writing – original draft (lead), writing – review and editing (lead). **José Jaime Zúñiga‐Vega:** conceptualization (equal), methodology (equal), writing – original draft (equal), writing – review and editing (equal). **Oscar Flores‐Villela:** resources (equal), writing – original draft (equal), writing – review and editing (equal). **Hibraim Adán Pérez‐Mendoza:** conceptualization (equal), supervision (equal), writing – original draft (equal), writing – review and editing (equal).

## Conflicts of Interest

The authors declare no conflicts of interest.

## Supporting information


Data S1.

## Data Availability

Morphological and life‐history trait database is available in Data S1.
